# *In-vitro* Optimization of Nanoparticle-Cell Labeling Protocols for *In-vivo* Cell Tracking Applications

**DOI:** 10.1038/srep15400

**Published:** 2015-10-28

**Authors:** Oshra Betzer, Rinat Meir, Tamar Dreifuss, Katerina Shamalov, Menachem Motiei, Amit Shwartz, Koby Baranes, Cyrille J. Cohen, Niva Shraga-Heled, Racheli Ofir, Gal Yadid, Rachela Popovtzer

**Affiliations:** 1 Gonda brain research center, Bar-Ilan University, Ramat Gan 52900, Israel; 2 Faculty of Engineering and the Institute of Nanotechnology and Advanced Materials, Bar-Ilan University, Ramat Gan 52900, Israel; 3 The Mina and Everard Goodman Faculty of Life Sciences, Bar-Ilan University, Ramat-Gan, 52900, Israel; 4 Pluristem Therapeutics Ltd., MATAM Advanced Technology Park #20, Haifa 31905, Israel

## Abstract

Recent advances in theranostic nanomedicine can promote stem cell and immune cell-based therapy. Gold nanoparticles (GNPs) have been shown to be promising agents for *in-vivo* cell-tracking in cell-based therapy applications. Yet a crucial challenge is to develop a reliable protocol for cell upload with, on the one hand, sufficient nanoparticles to achieve maximum visibility of cells, while on the other hand, assuring minimal effect of particles on cell function and viability. Previous studies have demonstrated that the physicochemical parameters of GNPs have a critical impact on their efficient uptake by cells. In the current study we have examined possible variations in GNP uptake, resulting from different incubation period and concentrations in different cell-lines. We have found that GNPs effectively labeled three different cell-lines - stem, immune and cancer cells, with minimal impairment to cell viability and functionality. We further found that uptake efficiency of GNPs into cells stabilized after a short period of time, while GNP concentration had a significant impact on cellular uptake, revealing cell-dependent differences. Our results suggest that while heeding the slight variations within cell lines, modifying the loading time and concentration of GNPs, can promote cell visibility in various nanoparticle-dependent *in-vivo* cell tracking and imaging applications.

Theranostic medicine is emerging as a new approach for promoting cell-based therapy, including stem cell therapy and immune cell therapy. Stem cells have broad applicability in fields such as oncology, cardiology, neurology and regenerative medicine^1^,^2^,^3^,^4^,^5^,^6^, due to their inherent biological properties, including their ability for self-renewal, differentiation into multiple cell types, and migration of transplanted cells^7^. Mesenchymal stem cells (MSCs) in particular, can be used as effective targeted therapy, as they exhibit homing capabilities to sites of injury and inflammation, exert anti-inflammatory effects, and can differentiate in order to regenerate damaged tissue^8^. Immune cell therapy shows great promise for treating autoimmune diseases and cancer^9^, and several recent clinical trials have successfully used tumor-specific cytotoxic T-cells, or adoptive T-cell therapy, for personalized medicine^10^,^11^,^12^,^13^. Interestingly, cancer cells have self-renewal regulation mechanisms similar to stem cells, and show extensive proliferation properties. Therefore, in addition to their use for *in vitro* studies of cancer and anti-cancer therapies^14^,^15^, cancer cells are widely used for general *in-vitro* cell research and as a model for regenerative medicine^16^.

Recent advances in nanotechnology offer an efficient platform for theranostic medicine. Engineered nanoparticles (NPs) loaded into cells serve as imaging contrast agents, enabling cell tracking in several imaging modalities such as computed tomography (CT), ultrasound (US) and magnetic resonance (MR) imaging. In addition, NPs conjugated to drugs can be used for targeted therapy when loaded in stem or immune cells, as the migratory properties of these cells make them massive delivery vehicles that increase NP transport to sites of injury^8^,^17^. Drug release from such hybrid systems can be regulated by various triggers, including external stimuli light^18^,^19^,^20^, ultrasonic irradiation and magnetic fields—thus minimizing side effects and improving treatment efficacy^21^.

The current study focuses on the process of loading cells with gold nanoparticles (GNPs), to be used in various theranostic applications. GNPs are well-known for their biosafety and long circulation times^22^,^23^. Moreover, they have a high degree of flexibility in terms of particle size, shape and functional groups for coating and targeting. GNPs can be quantitatively and sensitively detected *ex vivo* by atomic absorption methods, and serve as ideal contrast agents for *in vivo* CT imaging^24^,^25^. Therefore, GNPs have various therapeutic applications as drug carriers, biomarkers, biosensors and contrast agents. Surface modifications expand these applications by enabling GNPs to target specific sites on cell surfaces, organelles, the nucleus or extracellular matrix^26^. GNPs are also emerging as effective agents for *in vivo* cell tracking for cell-based therapy^27^,^28^. Recently, we developed a non-invasive, GNP-based CT imaging technique for tracking transplanted stem cells within the brain^28^ and tracking transduced T-cells in a melanoma bearing mouse model^29^.

However, a critical challenge in applying cell tracking and cell therapy with GNPs is to attain optimal efficacy of cell labeling, while verifying that the particles have no (or at least minimal) effect on cell function and viability. Several studies^30^,^31^,^32^ have examined the effect of GNP design – such as differences in material, size, shape and targeting agents – on cell uptake. In the current study we examined possible variations in GNP uptake resulting from time- and concentration-dependence of internalization, in different cell-lines. We used particles of the same size and coating (glucose coated 20 nm GNPs) to assess the efficiency of GNP uptake in three different cell-lines: A431 (cancer cells), PLX-PAD (mesenchymal-like stem cells) and T-cells (immune cells). We explored optimal GNP concentrations and incubation periods for particle loading into these different cell-lines, as well as the effect of GNP loading on viability, proliferation and biological activity of the cells.

## Results and Discussion

### GNP synthesis and characterization

**Figure 1:**
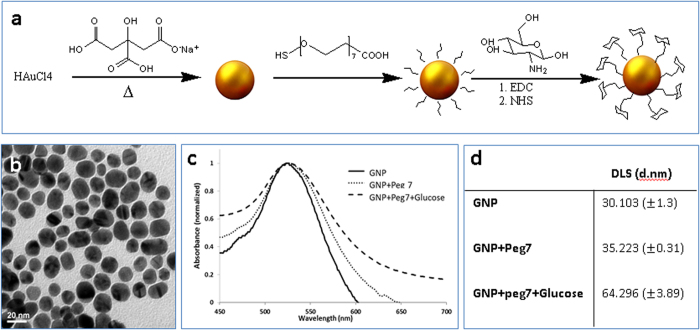
Characterization of GNPs. (**a**) Schematic diagram of the GNP synthesis process: GNPs were conjugated to the linker PEG7 (95%, Sigma-Aldrich, Israel Ltd) followed by covalent conjugation to glucose (GLU) (D-(+)-Glucosamine hydrochloride, Sigma-Aldrich, Israel Ltd.). (**b**) TEM image of 20 nm GNPs (scale bar 20 nm). (**c**) Optical properties of GNPs: UV-vis spectroscopy of bare GNPs, PEG7 coated GNPs and Glucose-PEG7 coated GNPs. (**d**) Mean hydrodynamic diameter obtained by DLS, at room temperature and at a scattering angle of 90°, at the various stages of GNP coatings. DLS measurements were carried out in triplicate. The significant differences obtained (both by DLS and UV-Vis spectroscopy) following each chemical step, demonstrates the efficiency of the chemical coating.

Based on a well-established procedure that was modified by our group^25^,^33^, 20 nm GNPs were synthesized (Fig. 1a). In brief, 520 ml 50% w/v of HAuCl4 mixed with 200 ml purified water. This mixture was heated until boiling, and then 4.04 ml sodium citrate was added. The solution was centrifuged until precipitation of nanoparticles and a clear suspension was obtained. For Glucose receptor targeting, which is a protein family found in most mammalian cells and a vital source of energy for all life^34^ and due to its stability and high cell-uptake rate, GNPs were coated with a layer of PEG7 (95%, Sigma-Aldrich, Israel Ltd.) linker. The PEG layer was covalently conjugated to Glucose-2 (2GF)(D-(+)-Glucosamine hydrochloride, Sigma-Aldrich, Israel Ltd.) by addition of EDC and NHS (200 ml of each chemical, Thermo Scientific, USA) to the solution. The solution was left to stir overnight in order to ensure the conjugation of the PEG layer to the Glucose. To purify the GNPs, the solution was centrifuged until obtaining a clear suspension. The particles were characterized using transmission electron microscopy (TEM), dynamic light scattering (DLS) and ultraviolet-visible (UV-vis) spectroscopy (Fig. 1b–d). The spherical gold nanoparticles were free from aggregation, and size variation was <4%. The efficiency of GNP coating was confirmed by the UV–vis Plasmon resonance shift and broadening (Fig. 1c), an expanded signal was observed following each layer coating, as the organic substance absorbs more energy from the irradiated light. Size distribution using DLS (Fig. 1d) also revealed the chemical coating changes. DLS showed a larger particle size (of 30.103 (+1.3) nm) as compared to the TEM images, due to the hydrodynamic diameter of the particles. All research experiments were conducted with those 20 nm glucose-modified nanoparticles in order to rule out uptake effects resulting from particle size and coating variations.

### Labeling cells with GNPs

First, we examined the feasibility of the uptake of GNPs by three different cell lines: Human squamous carcinoma cancer cells (A-431), Human immune cells (T-Cells) and placenta-derived mesenchymal-like adherent stromal cells (PLX-PAD). PLX-PAD are human placental expanded (PLX) mesenchymal-like adherent stromal cells produced by Pluristem Ltd., Israel, and are characterized by a high expression (≥93%) of typical Mesenchymal Stem Cell (MSC) markers CD105, CD90, CD73 and CD29 and lack of expression of hematopoietic, endothelial and trophoblastic-specific cell markers. In contrast to other known stem cell populations, PLX-PAD cells don’t differentiate into adipose or bone cells under standard differentiation conditions^35^. Nevertheless for brevity, the A-431, T-cells and PLX-PAD cells will be referred to in this manuscript, as cancer cells, immune cells and stem cells, respectively.

**Figure 2:**
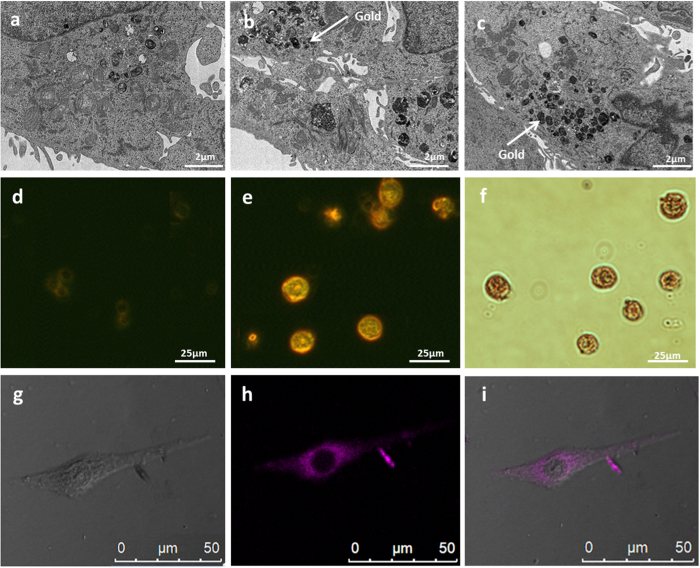
Efficiency of cell labeling with GNPs; microscopy images. (**a–c**) TEM imaging of a cancer cell. (**a**) cancer cell without GNPs. (**b,c**) cancer cell loaded with GNPs, the white arrow points an accumulation of well-defined GNPs inside endosomes. (**d,e**) dark field microscopy of stem cells (**d**) without GNPs. (**e**) loaded with GNPs (Yellow-red). (**f**) Bright field microscopy of stem cells loaded with GNPs. (**g–i**) representative confocal images of cancer cell after 30 min incubation with fluorescent-coated glucose-GNPs complex. (**g**) bright field image of the cell. (**h**) fluorescent-coated glucose-GNPs (purple). (**i**) Bright field combined images. Images were taken at the midsection of the cell. Sections were imaged using Leica TCS SP5 with Acousto-Optical Beam Splitter microscope.

The efficiency of cell-labeling was confirmed by obtaining transmission electron microscopy (TEM), confocal and dark-field microscopy images (Fig. 2). A total of 10^6^ cells were incubated with glucose coated GNPs (0.35 mg/mL) for one hour in 37 ^o^C. The TEM images clearly show nanoparticle internalization within endosomes. Confocal and dark field images distinctly demonstrate GNP uptake into all three cell lines.

In order to investigate the uptake mechanism, we ran a temperature dependence experiment, in which a total of 10^6^ cells were incubated with glucose coated GNPs (0.35 mg/mL) for half an hour in 37 ^o^C and 4 ^o^C. Research regarding uptake mechanisms of nanoparticles suggests that endocytotic cell uptake is driven by an energy-dependent, temperature-sensitive process^36^,^37^, and therefore, in order to prevent this internalization mechanism, the cells were incubated in 4 °C (and washed with ice-cold PBS).

**Figure 3:**
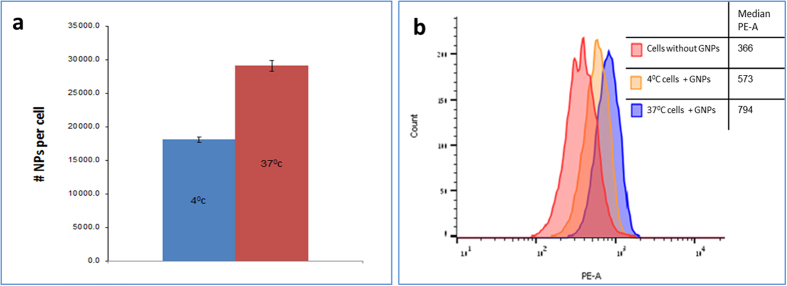
Uptake mechanism. (**a**) FAAS quantification of GNPs after half an hour incubation with cancer cells, under different temperature conditions (4 °C and 37 °C). Significant difference in gold amounts between the two temperatures (p < 0.01). (**b**) Representative histograms of GNP binding and uptake in cancer cells determined by flow cytometry (FACS). Red line: control cancer cells (without GNPs), orange line: cancer cells loaded with GNPs in 4 °C; blue line: cancer cells loaded with GNPs in 37 °C.

Results measured by analytical spectroscopy (Flame Atomic Absorption System—FAAS) revealed a clear reduction of the uptake at 4 ^o^C in comparison to the uptake at 37 ^o^C with a reduction of 38% (Fig. 3a). In addition, a flow cytometry (FACS) experiment that was performed under the same temperature conditions, demonstrated the difference between GNPs internalization within the cells and GNPs bound to the membrane (Fig. 3b). In natural glucose molecules the cellular uptake mechanism was found to be closely related to glucose transporters^38^,^39^,^40^,^41^. Interaction of GNPs with the glucose receptor may play a role in the uptake of GNPs through endocytosis^42^,^43^. The uptake process occurs in two steps: adsorption onto the membrane of the cell and then internalization by the cell^44^.

### Cell function following labeling with GNPs

**Figure 4:**
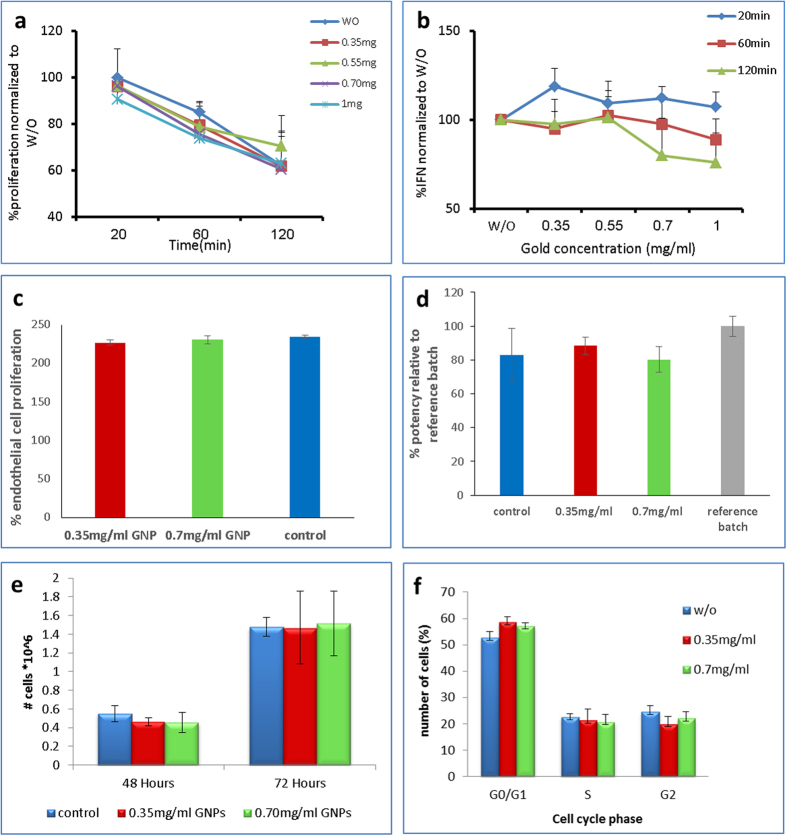
Cell proliferation and functionality assays for immune-cells (a,b), stem cells (c,d) and cancer cells (e,f). T-cells: (**a**) Proliferation assay. CFSE-labeled T-cells loaded with 0.35, 0.55, 0.70 and 1 mg/ml GNP concentrations, stimulated for 3 days and analyzed for CFSE dilution. Data shown as average percentage of proliferative cells, normalized to control (cells w/o GNPs) ± SEM. No significant differences observed between cells loaded with the different amounts of GNPs and controls (p > 0.05). (**b**) Functionality. T-cells loaded with 0.35, 0.55, 0.70 and 1 mg/ml GNP concentrations, co-cultured with positive target tumor cell line (888-A2) for 120 minutes. IFN-γ secretion (measured by ELISA) was normalized to control (cells w/o GNPs) ±SEM (n = 3) (p < 0.05, Student’s paired t-test). Cell-function was impaired only for high gold concentrations (0.75, 1 mg/mL) after 120 min of incubation (**c**,**d**) PLX-PAD cells. (**c**) Endothelial cell-proliferation assay. Cells loaded with 0.35 and 0.70 mg/ml GNP concentrations were grown for 48 h. Medium was changed to endothelial growth medium under hypoxic conditions for 24 hours before collection of conditioned medium (CM) (used to examine endothelial cell proliferation). Amount of viable cells after 4 days in culture with PLX-PAD derived CM was determined using the Alamar blue assay. (**d**) Cell adhesion assay. Following staining with 0.35 and 0.70 mg/ml GNP concentrations, Cells were left to adhere for 6 hours. Relative amount of viable adherent cells was determined using Cyquant. The adherence of cells was determined relative to the reference sample. No significant differences in endothelial cell proliferation and adherence were observed between CM derived from cells loaded with the different amounts of GNPs (p > 0.05). (**e**,**f**) A-431 cells. (**e**) Cell viability changes during 72 hours of A-431 cells loaded with 0.35 and 0.7 mg/ml GNP concentrations. Viable cells were identified by Trypan Blue-dye exclusion viability assay. Results presented as mean ± SEM (n = 3). (**f**) cell-cycle analysis. A-431 cells loaded with 0.35 and 0.70 mg/ml GNP concentrations were incubated for 48 h. Cells were then harvested, washed and fixed for 24 h. Data was analyzed using flow-cytometry. Results presented as mean ± SEM (n = 3). No significant differences in proliferation and viability were observed between cells loaded with the different amounts of GNPs (p > 0.05).

Using various assays, we next assessed the effect of GNP loading on viability, proliferation and biological activity such as metabolism and cell adhesion, of the different cell types at several time points. Representative results for each cell line are presented in Fig. 4 (for immune cells: CFSE proliferation and IFN secretion assays as a measure of cell viability and biological activity; for stem cells (represented by PLX-PAD): Cell adhesion assessment as a measure for cell viability and conditioned medium-induced endothelial cell proliferation to assess PLX-PAD secretion of angiogenic cytokines as a measure of biological functionality.^35^; for cancer cells: cell density and cell cycle; (additional tests can be found in Supplementary Figures). Results indicate minimal impairment of cell viability – cell proliferation remains unaltered after labeling with GNPs. In addition, the cells remained biologically and functionally active, with only a slight impairment to T-cells at higher GNP concentrations (0.70 & 1 mg/mL) and long incubation periods (120 min).

#### Optimizing the upload protocol for different cell types

To optimize cellular uptake of the GNPs, we measured two major factors that affect the total amount of gold per cell, namely, the time course of the upload process and GNP concentration.

##### Time course of GNP uptake into cells

**Figure 5:**
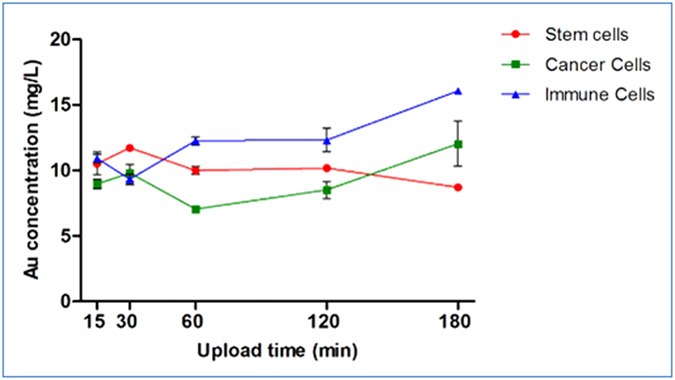
Cellular uptake of GNPs as a function of incubation time. All three cell lines were incubated with GNPs (0.35 mg/ml) for increasing time periods, up to 3 hours. The actual amount of gold uptake was quantified using FAAS analysis. Results presented as mean ± SEM (n = 3).

**Figure 6:**
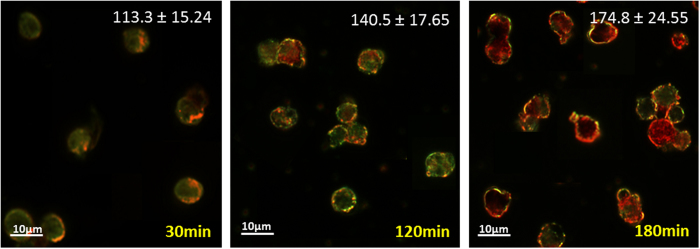
Microscope imaging of GNP uptake into immune cells at increasing incubation periods. Dark field microscopy of T-cells incubated with GNPs (0.35 mg/ml) for 30, 120 and 180 minutes. Cells colored green gradually change to yellow and then red, according to the GNP concentration within cells. The GNP density values within cells are presented in each image (top; Grey value range: 0–255, obtained by Image J).

We incubated all three types of cells with 0.35 mg/ml GNPs and analyzed the average amount of uptake at different time points (15, 30, 60, 120 and 180 min) using Flame Atomic Absorption Spectroscopy (FAAS, SpectrAA140; Agilent Technologies) (Fig. 5). It appears that for all cell types, the capacity for gold uptake stabilizes after 1 hour of incubation. Immune cells and cancer cells show a slight increase in gold uptake after 2 hours, while the stem cells show a slight decrease. Figure 6 displays dark field microscopy images of T-cell density changes observed over 3 hours of incubation with GNPs (density was measured by grey values using ImageJ 1.48V (National Institutes of Health, USA). In the current study, we incubated the cells only up to 3 hours in order to prevent cell adherence to the wells, which would require an additional detachment procedure. As the purpose of this study was to optimize labeling of cells for immediate *in-vivo* transplantation, the protocol had to enable rapid GNP uptake, with minimal handling of cells. Thus, our results suggest that the optimal labeling time is between 1 hour (at which the amount of gold per cell stabilizes) and 3 hours. This allows selection of specific labeling periods according to the particular goals and needs of individual studies.

##### Effect of GNP concentration on uptake into cells

**Figure 7:**
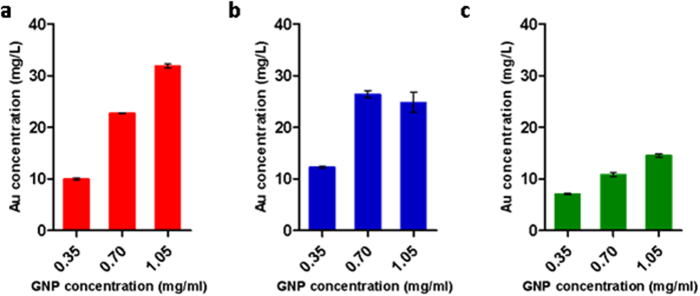
Cellular uptake of GNPs as a function of gold concentration. Each cell type was incubated with GNPs at three different concentrations (0.35, 0.7, and 1.05 mg/ml), for 1 h. The actual amount of gold uptake was quantified using FAAS analysis (significant difference between different gold amounts (p < 0.001), except for high GNP concentration in T-Cells (p > 0.05)). (**a**) Stem cells. (**b**) Immune cells. (**c**) Cancer cells. Results presented as mean ± SEM (n = 3).

**Figure 8:**
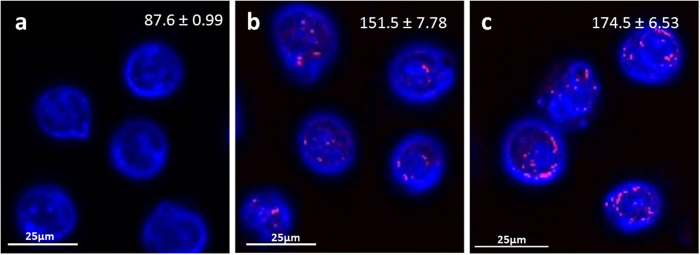
Microscope imaging of GNP uptake into cancer cells as a function of gold concentration. Dark field microscopy of A-431 cells (blue) incubated for 1 h with GNPs (pink) at three different concentrations (**a**) no GNPs, (**b**) 0.35 mg/ml. (**c**) 0.7 mg/ml. The spectrum of the GNPs was obtained after reduction of the background signal (cells without GNPs) from the total reflectance signal (cells with GNPs). Density value of cells is presented in each image (top; Grey value range: 0–255, obtained by Image J).

We then examined whether gold concentration affects the total per-cell uptake of gold. As previously described by See *et al.*^45^ who tested uptake of Nanobioconjugates into various cell lines, the gold concentration plays a significant role in affecting the number of particles internalized within a cell. Thus, the three cell-lines were incubated with 0.35 mg/ml, 0.70 mg/ml or 1.05 mg/ml GNPs for 1 hour, and the average amount of GNP uptake was analyzed using FAAS (Fig. 7). In addition, dark-field microscopy images confirmed the differences in uptake resulting from GNP concentrations (Fig. 8). We found that the higher gold concentration significantly increased the amount of gold uptake per cell, although differences between cell-lines were clearly noticeable (Fig. 7). In immune cells, uptake increased as the gold concentration increased from 0.35 to 0.70 mg/ml, then stabilized, and even slightly decreased, with the further increase to 1.05 mg/ml. For stem cells, gold uptake significantly and steadily increased with the increase in concentration, and cancer cells likewise revealed a constant, though smaller, increase in gold uptake. Cancer cells and stem cells have been shown to be less sensitive to gold concentration. In addition, since they are bigger in volume than immune cells which are primary cells, and more resistant to cell death^46^,^47^, they can contain a greater number of nanoparticles without detriment to their function and viability. Therefore, our results imply that cell-dependent characteristics should be taken into account while developing cell-tracking protocols.

## Conclusions

In the present study, we investigated GNP uptake into three different representative and broadly used cell lines; Human squamous carcinoma cancer cells (A-431), Human immune cells (T-Cells) and placenta-derived mesenchymal-like adherent stromal cells (PLX-PAD). Although throughout this study, the cells were referred to as stem, immune and cancer cells, it is important to note that there may be considerable differences between each cell line within these groups.

We found that GNPs effectively labeled the various cell lines, with minimal impairment of cell viability and functionality. These results confirm that GNPs can be safely used for labeling and real-time prolonged tracking of cells. Moreover, the finding that the amount of GNPs taken up by cells stabilizes after a short period of time (1 hour), allows for selection of optimal labeling periods for specific studies. We found that GNP concentration had a significant impact on cellular uptake, and revealed more cell-dependent differences than incubation time.

Fundamental *in vitro* studies have established that physiochemical-dependent NP uptake cannot be overlooked when designing NP-based systems for biomedical applications^31^,^48^. These findings are augmented by our results, which reveal that the specific cell line utilized should be taken into consideration along with NP properties. Moreover, our results allow selection of specific labeling periods and GNP concentrations, based on the particular cell line used, as well as the goals and needs of each individual research. For studies requiring maximum NP uptake into the cells, GNP concentration should be dramatically increased. For the purpose of gaining visibility while maintaining long-term viability, a lower concentration may be used, especially with more vulnerable cell types such as immune cells. Future studies should elucidate the effect of specific cell-dependent characteristics on GNP uptake, to promote and improve the design of GNP systems for biomedical applications.

In summary, our results suggest that modifying the loading time and concentration of GNPs, while heeding the slight variations within cell lines, can promote cell visibility in various nanoparticle-dependent cell tracking and imaging applications.

## Methods

**GNP Synthesis**: A total of 0.414 mL of 1.4 MHAuCl4 solution in 200 mL of water was added to a 250 mL single-neck round-bottom flask and the solution was stirred in an oil bath on a hot plate until it boiled. Then, 4.04 mL of a 10% sodium citrate solution (0.39 M sodium citrate tribasic dihydrate 98%, Sigma CAS 6132-04-3) was quickly added. The solution was stirred for 5 min, and then the flask was removed from the hot oil and placed aside until it cooled.

**GNP Conjugation**: To prevent aggregation and stabilize the particles in physiological solutions, PEG7 (95%, Sigma-Aldrich, Israel Ltd.) was absorbed onto the GNPs First, the solution was centrifuged to dispose of excess citrate. PEG7 solution (2.26 × 10^3^ g) was then added to the GNP solution, and the mixture was stirred overnight and subsequently centrifuged. Next, excess EDC (N-ethyl-N -(3-(dimethylamino)propyl)carbodiimide (1.87 × 10^−3^ g) and NHS (N-hydroxysuccinimide) (Thermo Fisher Scientific, Inc., Rockford, IL) (2.12 × 10^3^ g) were added to the solution, followed by addition of glucose-2 (2GF)(D-(þ)-glucosamine hydrochloride, Sigma-Aldrich, Israel Ltd.) (1.75 × 10^3^ g). NHS and EDC form an active ester intermediate with the –COOH functional groups, which can then undergo an amidation reaction with the glucose-NH_2_ group. Glucosamine molecule C-2 (2GF-GNP): D-(þ)-glucosamine hydrochloride (3 mg; Sigma-Aldrich) was added to the activated linker-coated GNPs.

### Cell Isolation and Expansion

**PLX-PAD cells preparation**: The production of PLX-PAD is performed in a state-of-the-art clean room facility according to good manufacturing practice (GMP) regulations. The production process is composed of several major steps that include receipt of the placenta, recovery and processing of adherent stromal cells, growth of the cells in tissue culture flasks [two-dimensional (2-D) cultures] and harvest and storage of the cells in liquid nitrogen as 2-D cell stock (2DCS). The 2DCS is considered to be an in-process intermediate product and is tested for sterility, mycoplasma, immunophenotype and viability. Upon meeting 2DCS release specifications, the appropriate amount of 2DCS is thawed, washed and seeded onto carriers in bioreactors for further expansion in three-dimensional (3-D) culture. After 1–2 weeks of growth in the bioreactors, the cells are harvested and cryopreserved in liquid nitrogen as PLX-PAD.

**A431 cells preparation**: 15 Petri dishes were seeded with 500,000 A431 Human neck-head cancer cells for each dish. 10 ml DMEM medium was added for each one of the dishes. The cells were incubated at 37 °C for 48 hours without medium replacement.

**T-cells preparation**: Primary human T-Lymphocytes were cultured in BioTarget medium (Biological Industries, Beth Haemek, Israel) supplemented with 10% heat-inactivated FBS and 300 IU/ml IL-2 and maintained at 37 °C and 5% CO2.

**Sample preparation for TEM**: 4 Petri dishes were seeded with 500,000 A431 Human neck-head cancer cells for each dish. 5 ml DMEM medium (without glucose) was added for each one of the dishes. The cells were incubated at 37 °C for 48 hours without medium replacement. DMEM medium was removed. Cells were fixed with 2 ml glutaraldehyde per dish was done. The cells were incubated for 1 h in room temperature followed by scraping of the cells with a rubber policeman into Eppendorf tubes. Cells were then washed with Cacodylate buffer, 1% Osmium, 70%, 90%, 100% Alcohol washing twice, 1:0, 3:1, 1:3 Propilen oxide:AGAR washing.

**Cell binding study**: Cells (0.5 × 10^6^) were cultured in 5 mL of glucose-free DMEM medium containing 5% FCS, 0.5% penicillin, and 0.5% glutamine. GNPs were then added in excess (one million particles per cell). The cells were then incubated at 37 °C for the relevant duration. After incubation, the medium was washed twice with PBS, followed by trypsin treatment; the cells were centrifuged twice (5 min in 1000 rpm) to wash out unbound nanoparticles.

**Membrane binding study**: Cells (0.5 × 106) were cultured in 5 mL of glucose-free DMEM medium containing 5% FCS, 0.5% penicillin, and 0.5% glutamine. GNPs were then added in excess (one million particles per cell) at 4 °C to block endocytic uptake. Incubation time was set to 15 minutes, to avoid cell internalization by direct membrane penetration. All solutions used in the cell surface binding experiments were pre-cooled on ice to maintain experimental conditions strictly at 4 °C. Cells were kept on ice for ~10 min prior to experiment to inhibit endocytosis. After incubation, the medium was washed twice with ice-cold PBS, followed by trypsin treatment; the cells were centrifuged twice (5 min in 1000 rpm) to wash out unbound nanoparticles. The membrane binding was tested using flow cytometric analysis and is expressed as mean intensity which represents the GNPs fraction that is resistant to washing and thus tightly bound to the cell surface.

**Confocal microscopy experiment**: Fluorescent coated (Rhodamine B, Sigma, Israel) GNPs were incubated with A431 cells for 30 min at 37 °C. The cells were subsequently washed three times in PBS prior to confocal imaging using Leica TCS SP5 with Acousto-Optical Beam Splitter microscope to acquire fluorescent and bright field images.

**Flow cytometry analysis of T-cell proliferation and viability**: Immunofluorescence, analyzed as the relative log fluorescence of live cells, was measured using a CyAn-ADP flow cytometer (Beckman Coulter, Brea). Approximately 1 × 105 cells were analyzed. For cell proliferation assay, T-cells were labeled with 1 μM CFSE (eBioscience, San Diego, CA) for 6 min and then cultured in the presence of 0.1 mg/ml plate-bound OKT3 (eBioscience, San Diego, CA). Cells fluorescence was analyzed by flow cytometry. Cell viability assays were performed as follow - after T-cells labeling with different amounts of gold (0.38, 0.57, 0.76 and 1 mg/ml), cells were labeled with 1 μM propidium iodide (PI) (Sigma-Aldrich, Israel) to assess the ratio of cell death. Samples were analyzed by flow-cytometry. For the different analysis procedures, cells were incubated in buffer made of PBS, 0.5% BSA, and 0.02% sodium azide. The results of cytokine secretion were compared using a paired two-tailed Student’s t test. p-values below 0.05 were considered significant.

**PLX Endothelial cell proliferation assay**: PLX –PAD cells were stained for 1 hour with different GNP concentrations (0, 0.35 and 0.70 mg/ml). Following staining with GNPs, PLX cells are seeded according to viable cell count in 6 well plates in full DMEM medium. The next day the medium is changed to serum free endothelial growth media and incubated for 24 hours in hypoxic conditions. Endothelial cells are then grown for 4 days in the presence or absence of the different PLX derived CM (diluted 1:1 with fresh endothelial growth medium containing FBS- to avoid growth inhibition due to medium consumption and lack of FBS). The amount of viable cells is determined at the end of this period using the Alamar blue assay. The results show proliferation of endothelial cells exposed to different PLX derived CM in comparison to cells grown in endothelial growth medium with the same FBS concentration.

**PLX-PAD cell adhesion assay**: According to total cell count at the end of the cell staining procedure, 20,000 cells are seeded in 96 well plates. Cells are placed in an incubator and left to adhere for 6 hours. After 6 hours, unattached cells are washed and the relative amount of viable adherent cells is determined using Cyquant. The reference sample, which is thawed, suspended in full DMEM media, counted and seeded (without any further manipulation) is considered 100% adherence potency. The adherence of cells that underwent the staining procedure (with or without GNPs) was determined relative to the reference sample.

**A-431 Cell cycle analysis**: To test the survival of A431 cells, tumor cells were loaded with different amounts of gold and incubated at 37 °C for 48 h. Cells were then harvested and washed twice in cold PBS. Following the wash, cells were fixed for 24 h at 4 °C, using 4 ml cold ethanol (−20 °C, 70%). For PI staining and flow cytometric analysis, fixed cells were washed and centrifuge in 500 g, for 5 min. Cell pellet then was resuspended in 400 μl PBS supplemented with 8 μl RNAse (1 μg/ml) and 4 μl PI (2 μg/ml). Samples were incubated for 10 min in the dark, before analyzed by flow-cytometry.

**Flame Gold Analysis**: The cells were melted with aqua regia acid, a mixture of nitric acid and hydrochloric acid in a volume ratio of 1:3. The samples (3 samples for each experimental condition) were then evaporated, filtered and diluted to a final volume of 10 mL. Au lamp was used in order to determine the gold concentration in the samples. A calibration curve with known gold concentrations was prepared (commonly: 0.1, 1, 2 and, 5 mg/mL). Gold concentration in each sample was determined according to its absorbance value with correlation to the calibration curve. Each sample was analyzed in triplicate and averages and standard deviations were taken.

## Additional Information

**How to cite this article**: Betzer, O. *et al. In-vitro* Optimization of Nanoparticle-Cell Labeling Protocols for *In-vivo* Cell Tracking Applications. *Sci. Rep.* **5**, 15400; doi: 10.1038/srep15400 (2015).

## Supplementary Material

Supplementary Information
