# Supplementary material for: In-vitro Optimization of Nanoparticle-Cell Labeling Protocols for In-vivo Cell Tracking Applications
Source: Sci Rep. 2015 Oct 28;5:15400. doi: 10.1038/srep15400 (PMC4623670; doi:10.1038/srep15400)
Supplement: Supplementary Information [file srep15400-s1.pdf]

In-vitro Optimization of Nanoparticle-Cell Labeling Protocols for In-vivo Cell Tracking Applications

Oshra Betzer [1,2*], Rinat Meir[2]*, Tamar Dreifuss[2], Katerina Shamalov[3], Menachem Motiei[2], Amit Shwartz[3], Koby Baranes[2], Cyrille J. Cohen[3], Niva Shraga-Heled[4], Racheli Ofir[4], Gal Yadid[1,3] and Rachela Popovtzer[2]**

Supplementary figures:

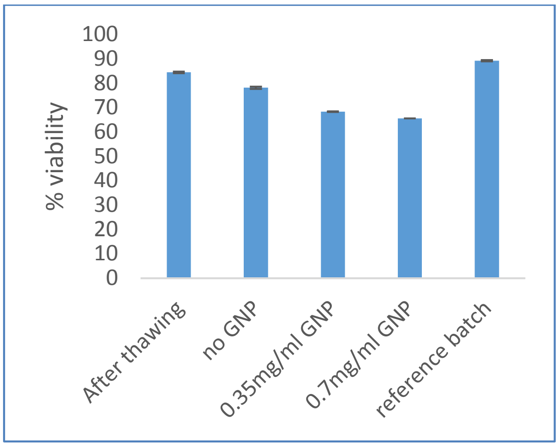

Figure 1: PLX-PAD cell viability assessment by Vi-Cell.

Cell viability by Vi-Cell

Cells were thawed in 37°C water bath and diluted immediately in warm full DMEM medium (containing 10% FBS). Cells were then centrifuged (1200RPM, 10 min, 4°C). Cell pellets were suspended in saline and counted using Vi-Cell. According to cell counts, cells were suspended to 0.5*106 cells/ml in saline in 50ml tubes, GNPs were added to desired concentration (0, 0.35, 0.7 mg/ml). Cells were incubated with GNPs in closed tubes for 1hr at 37°C with gentle agitation (50rpm).After staining cells were centrifuged (200g, 5min, RT) and washed twice with saline (same volume as in incubation). After two washes cells were suspended in full DMEM medium to an estimated concentration of ~1*106 viable cells/ml. After suspension cells were counted again in Vi-Cell (results are represented in figure 1a. Reference samples were thawed just before seeding for experiments. Cell viability and number were determined using Vi-Cell, before and after cell staining as described above. It can be seen that the percentage of viable cells is decreased by 10-40% when staining with GNPs, in a concentration dependent manner (cells stained with a higher concentration of GNPs are less viable).

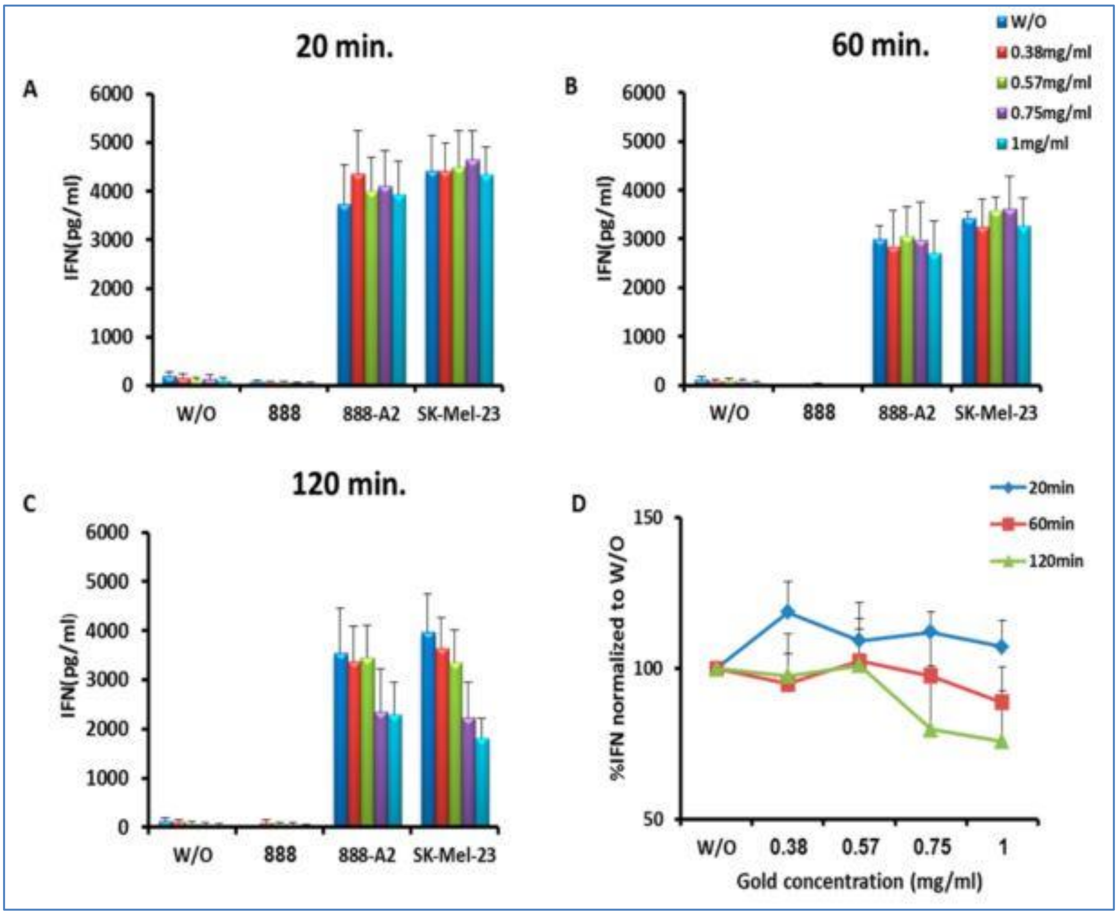

Figure 2: Function of GNP-loaded F4-transduced human T-cells. TCR-transduced primary human T-cells were loaded with different concentrations of gold (0.38, 0.57, 0.75 and 1 mg/ml) and co-cultured with multiple melanoma lines, as indicated. IFN-γ secreted in the co-culture supernatant was measured by ELISA. Data shown as average secretion from three different donors. Secretion data for each time point (20, 60 and 120 min) is displayed separately (A, B and C, respectively). D. IFN secretion of F4-T-cells after co-culture with positive target tumor cell line (888-A2) was normalized to control (cells without (w/o) GNPs; $p<0.05$, Student's paired t-test). Results presented as mean ± SEM (n=3).

Function of GNP-loaded F4-transduced human T-cells

To test their biological activity, GNP-loaded primary human T-cells were co-cultured with either HLA-A2+/MART-1+ 888-A2, SK-MEL-23 or 888 (HLA-A2-/MART-1+, negative control) human melanoma cell lines for 18 hrs. T-cell function assessed by IFNg secretion in cells pre- or post-loaded with GNPs at different time points revealed a moderate reduction in cytokine secretion as a function of time (15.2% less IFN secretion for 60 min loading time, and 21.8% less IFN secretion for the 120 min loading time, as compared with the 20 min loading time of T-cells co-cultured with 888-A2; Figure 2). Furthermore, comparison of multiple gold loading concentrations showed that cell function was impaired mostly for the higher gold concentrations (0.75 mg/ml and 1 mg/ml), after 120 min of incubation (21% and 25% less IFN secretion in co-culture with 888-A2 cells, respectively, compared to pre-loaded T-cells

Figure 2C-D). No significant cytokine secretion was noted in co-cultures with control 888 melanoma line or pre-loaded -loaded T-cells, for any of the incubation time points or gold concentrations (Figure 2A-C).

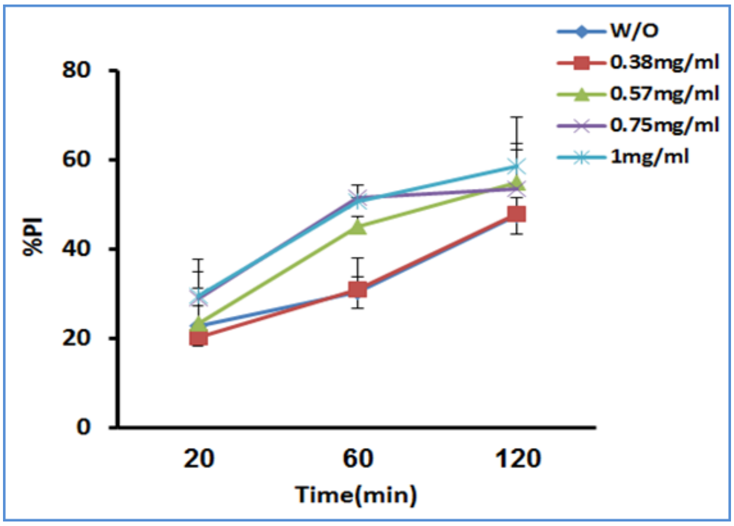

Figure 3: T cells viability assay. F4-expressing T-cells were loaded with different GNP concentrations (0.38, 0.57, 0.75,and 1 mg/mL)and cultured for 20, 60, or 120 min. PI was added to the supernatant, and the cells were analyzed by flow cytometry. Data is shown as mean percentage of positive PI cells from three different donors. Results presented as mean SEM (n = 3).

Determination of T cells death

We additionally evaluated the effect of GNPs on T-cell viability (Figure 3). Propidium iodide (PI) was added to the gold-loaded cells, and PI positivity was assessed by flow cytometry. While there was no significant difference in cell viability for the different gold concentrations at 20 min loading time, increased incubation intervals resulted in a higher loss of viability, especially as the concentration of gold increased (1.5-fold more cell death for 60 min and more than 2-fold for 120 min, compared to the proportion of cell death for 20 min incubation).
